# Candidemia Surveillance and Impact on Non-neutropenic Critically Ill Patients

**DOI:** 10.7759/cureus.73155

**Published:** 2024-11-06

**Authors:** Lisa Hall Zimmerman, Heather Dolman, Janie Faris, Linda Park, Ryan Mynatt, William B Zimmerman, Alfred E Baylor, James Tyburski, Robert F Wilson

**Affiliations:** 1 Department of Pharmaceutical Services, William Beaumont University Hospital, Royal Oak, USA; 2 The Michael and Marian Ilitch Department of Surgery, Wayne State University School of Medicine, Detroit, USA; 3 Department of Surgery, Detroit Receiving Hospital, Detroit, USA; 4 Department of Pharmacy, Parkland Health and Hospital System, Dallas, USA; 5 Department of Pharmacy, Detroit Receiving Hospital, Detroit, USA; 6 Department of Osteopathic Surgical Specialties, Michigan State University, East Lansing, USA

**Keywords:** anti-infective agents, candidemia fungal, critical care, id critical care, surgical critical

## Abstract

Background: Candidemia is a common pathogen in critically ill patients and has a significant negative impact on morbidity and mortality. Risk factors linked with candidemia are reported in the literature. We evaluated the risk factors associated with candidemia in critically ill patients on mortality rates, including the impact of delayed or inadequate antifungal therapy (IAAT).

Methods: This retrospective study evaluated non-neutropenic critically ill adult patients with candidemia for six consecutive years. Antifungal therapy was evaluated for the following: the correct dose based on the *in vitro* activity against *Candida *species identified on culture, the time interval from culture positivity to the initiation of antifungal therapy, and the duration of antifungal therapy. Adequate antifungal therapy (AAT) was defined as the initial antifungal agent administered to the patient with *in vitro* activity against *Candida *species identified on culture using the correct dose, time of initiation, and duration of therapy. IAAT was determined if the antifungal did not have *in vitro* activity against the *Candida *species identified on culture with the initial incorrect dose.

Results: In the 91 critically ill patients evaluated with documented candidemia, the mean age was 57±16 years, the mean Acute Physiology and Chronic Health Evaluation II (APACHE II) score was 25±9, and the overall mortality rate was 38%. Patients with the following risk factors for candidemia had an increased mortality: use of mechanical ventilation (35 (100%), p<0.001), vasopressor therapy (28 (80%), p<0.001), end-stage renal disease (ESRD) (11 (31%), p<0.001), and ≥ 2 organ failure (23 (65%), p=0.002). Mortality was also more likely in patients who received IAAT: 16 (64%) IAAT vs. 19 (29%) AAT, p=0.001.

Conclusions: In critically ill patients with risk factors associated with candidemia, AAT is important when candidemia is suspected. This study found that *C. glabrata* was more likely isolated in patients with ESRD, vasopressor therapy for hemodynamic support, high APACHE II scores, and ≥ 2 organ dysfunction.

## Introduction

Candidemia causes 10-15% of nosocomial bloodstream infections in critically ill patients and is associated with mortality rates of 35-67% [1-3]. Recent literature indicates that inadequate antifungal therapy (IAAT) increases mortality rates associated with the related increase in infections by non-*Candida albicans* *(C. albicans) * species, especially *C. glabrata* [4-6].

Surveillance culture data from our institutions from 1997 to 2002 demonstrated a decrease in the frequency of *C. albicans *bloodstream infections in non-neutropenic patients from 50% to 40%, with a concomitant rise in *C. glabrata* cultures from 13% to 27% [4]. This shift in species distribution is particularly problematic due to *C. glabrata*'s inherent resistance to fluconazole, necessitating treatment with an echinocandin. These data were presented in part in abstract form during the 33rd Annual Meeting of the Surgical Infection Society in Las Vegas, Nevada. The objective of this study was to evaluate the risk factors associated with candidemia in critically ill patients on mortality rates, including the impact of delayed or IAAT. The secondary outcomes were to determine the risk factors associated with mortality and risk factors for *C. glabrata* isolation on culture.

## Materials and methods

This retrospective cohort study, approved by the institutional review board (IRB) at Wayne State University (108610MP4E), was conducted in a university-affiliated Level 1 trauma center. Patients were included if they were ≥18 years of age, admitted to the intensive care unit (ICU), and had at least one positive blood culture for *Candida* species from May 2006 to June 2012. Patients with a current clinical diagnosis of neutropenia were excluded to minimize bias regarding the primary outcome. Data collected included baseline demographics and clinical outcomes, including mortality, duration of mechanical ventilation, organ failure, and use of hemodynamic support.

Adequate antifungal therapy (AAT) was defined as the initial administration of an antifungal agent with *in vitro* activity against the *Candida* species isolated. An azole with the appropriate dose and duration of therapy was considered AAT for all *Candida* species except *C. krusei *and *C. glabrata. *An echinocandin (micafungin or anidulafungin) with the appropriate dose and duration of therapy was considered adequate for all *Candida* species except *C.* *parapsilosis.* During the study period, the study site did not have a protocolized approach (no established inclusion and exclusion criteria) for patients to initiate empiric antifungal therapy. Empiric therapy was initiated when blood cultures were identified as positive for yeast on culture. The time to AAT was defined as the time from obtaining the blood culture to the administration of an adequate antifungal medication.

The severity of illness was evaluated using the Acute Physiology and Chronic Health Evaluation II (APACHE II) [7]. Additionally, the use of intravascular catheters, parenteral nutrition, previous antimicrobial exposure, and steroid therapy were collected for the identification of risk factors for candidemia.

Data are presented as either mean ± SD or number (%). Statistical analysis was performed using IBM SPSS Statistics for Windows, Version 26 (Released 2019; IBM Corp., Armonk, New York). Pearson's X^2^ was used for univariate analysis with categorical data, Student's t-test for parametric data, and Mann-Whitney U for non-parametric continuous data as appropriate. A p-value of <0.05 was considered statistically significant.

## Results

During the study period, 91 patients had documented candidemia, and four of these patients had two species of *Candida* species on culture. The in-hospital mortality rate was 38%, with AAT being administered to 66 (73%) patients and 25 (27%) receiving IAAT. The mean APACHE II score was 25±9, and a central venous catheter was the assumed source of candidemia in 84% of patients (Table 1).

**Table 1:** Patient demographics ^#^data expressed as mean±SD; *N(%) APACHE II: Acute Physiology and Chronic Health Evaluation; ICU: intensive care unit; COPD: chronic obstructive pulmonary disease; TBSA: total body surface area

Characteristic	N=91
Age (years)^#^	57±16
Gender, male*	49 (54%)
Weight (kilogram)^#^	77±25
African American*	62 (68%)
APACHE II^#^	25±9
Intensive care unit and reason for admission	
Medical ICU	45 (50%)
Pneumonia	35 (39%)
COPD exacerbation	10 (11%)
Surgical/trauma ICU	28 (30%)
Abdominal pain	23 (25%)
Trauma, penetrating	5 (5%)
Burn ICU, >10% TBSA	15 (17%)
Neuro/neurosurgical ICU, ischemic stroke	3 (3%)
Past medical history	
Hypertension	58 (64%)
Diabetes mellitus	25 (27%)
Malignancy	16 (18%)
Coronary artery disease	12 (13%)

There were 95 *Candida* species found in 91 patients with *C. albicans* 38 (40%), *C. glabrata* 26 (27%), and *C. parapsilosis* 18 (19%), making the majority of isolates. Overall, a prolonged ICU length of stay of≥14 days occurred in 65 (69%) patients. Patients who developed candidemia were older (age >55 years) with a mean age of 57±16 years and were in the hospital a mean of 19±18 days before the onset of candidemia.

The 25 patients receiving IAAT were more likely to have in-hospital mortality, 18 (64%) IAAT vs. 19 (28%) AAT, p=0.001 (Table 2). Mortality was higher in patients with two or more organ dysfunction, 31/50 (62%) vs. those with less organ failure, 4/41 (10%), p<0.0001. In addition, patients with end-stage renal disease (ESRD) requiring dialysis, 11/13 (85%), were more likely to have in-hospital mortality (p=0.0003) (Table 3).

**Table 2:** Inadequate antifungal therapy ^N(%); p-value<0.05 is considered significant AAT: adequate antifungal therapy; IAAT: inadequate antifungal therapy

*Candida* Species^	All Species N=95	AAT N=66	IAAT N=25	P-value
Candida albicans	38 (40)	12 (35)	3 (75)	0.28
Candida glabrata	26 (27)	3 (43)	12 (63)	0.40
Candida parapsilosis	18 (19)	1 (7)	2 (66)	0.05
Candida tropicalis	12 (13)	3 (30)	1 (50)	0.99
Candida kefyr	1 (1)	1 (100)	0 (0)	-
Characteristics^	Patients N=91	AAT N=66	IAAT N=25	P-value
Overall in-hospital mortality	27	19 (28)	18 (64)	0.001
In-hospital mortality with mechanical ventilation	35	19 (29)	16 (64)	0.003
In-hospital mortality with vasopressors	28	14 (21)	14 (56)	0.002
In-hospital mortality with mechanical ventilation and vasopressors	28	14 (21)	14 (56)	0.002

**Table 3:** Factors impacting mortality ^N(%); p-value<0.05 is considered significant ESRD: end-stage renal disease; APACHE: acute physiology and chronic health evaluation; AAT: adequate antifungal therapy

Characteristics^	Patients N=91	Survival N=56	Non-survival N=35	P-value
Mechanical ventilation	76 (83)	41 (73)	35 (100)	<0.001
Use of vasopressors	48 (53)	20 (36)	28 (80)	<0.001
ESRD	13 (14)	2 (3)	11 (31)	<0.001
Inadequate antifungal therapy	25 (28)	9 (18)	16 (46)	0.002
1 organ failure	28 (31)	24 (43)	4 (11)	0.002
≥2 organ failure	41 (45)	18 (32)	23 (65)	0.002
APACHE II ≥30	25 (28)	10 (18)	15 (43)	0.009
Time AAT >72 hours	45 (49)	22 (39)	23 (65)	0.01
Candida glabrata	23 (25)	10 (18)	13 (37)	0.04
Severe sepsis	68 (74)	38 (68)	30 (86)	0.05
Age <50 years	26 (29)	20 (36)	6 (17)	0.05

Overall, the incidence of *C. glabrata* candidemia was 26/95 (27%). In older patients (age ≥ 60 years), the incidence of *C. glabrata* increased to 58%. Other factors associated with *C. glabrata *were APACHE II > 25 (18 (69%) *C. glabrata* vs. 32 (46%) non-*C. glabrata*, p=0.04) and a history of diabetes (11(42%) *C. glabrata* vs. 14 (20%) non-*C. glabrata*, p=0.03) (Table 4).

**Table 4:** Candida glabrata risk factors *mean±SD; ^N(%); p-value<0.05 is considered significant APACHE: acute physiology and chronic health evaluation; IAAT: inadequate antifungal therapy;  IJ: internal jugular; AAT: adequate antifungal therapy

Characteristics	*C. glabrata* N=26	Non-*C. glabrata* N=69	P-value
Age, years*	65.27±15.0	54.2±16.05	0.003
APACHE II score*	28.0±9.3	24.2±8.6	0.06
APACHE II >25^	18 (69%)	32 (46%)	0.04
IAAT with APACHE II >25^	14 (54%)	7 (10%)	<0.001
Diabetes mellitus^	11 (42%)	14 (20%)	0.03
Nursing home patient^	6 (23%)	6 (9%)	0.06
IAAT^	19 (73%)	9 (13%)	<0.001
Time to AAT ≥ 48 hours or death before antifungals^	22 (85%)	41 (59%)	0.02
Vasopressor therapy^	18 (69%)	32 (46%)	0.04
Age ≥60 years^	15 (58%)	21 (30%)	0.01
IJ and hemodialysis catheter present	8 (31%)	10 (14%)	0.07
In-hospital mortality^	15 (58%)	22 (32%)	0.02
Mortality with mechanical ventilation^	15 (58%)	22 (32%)	0.02
Mortality with APACHE II >25^	11 (42%)	15 (22%)	0.04
Time from positive culture to AAT, hours*	87.5±50.6	64.8±48.2	0.07
Admit to onset of fungemia, days*	14.5±18.3	20.7±17.5	0.14

In addition, IAAT occurred more with *C. glabrata* (19 (73%) vs. 9 (13%) non-*C. glabrata*, p<0.001). Patients with *C. glabrata* were more likely to require vasopressors for hemodynamic support (18 (69%) *C. glabrata *vs. 32 (46%) non-*C. glabrata*, p=0.04). In patients receiving AAT, the time to AAT was longer for patients with *C. glabrata* (87.5±50.6 hours *C. glabrata* vs. 64.8±48.2 non-*C. glabrata*, p=0.07).

Interestingly, 34 (37%) of candidemia patients had concomitant bacteremia with either a Gram-positive cocci (N=19), Gram-negative bacilli (N=12), or both (N=3). Concomitant bacteremia was common in patients with age ≥ 60 years (18 (53%) bacteremia vs. 17 (30%) no bacteremia, p=0.02), with a femoral venous catheter (10 (29%) bacteremia vs. 7 (12%) no bacteremia, p=0.04), and with an APACHE II score ≥25 (23 (68%) bacteremia vs. 25 (44%) no bacteremia, p=0.01).

Overall, the mean time to AAT was 67±49 hours. However, mortality was increased when the time to AAT was ≥72 hours (39% survivors vs. 65% non-survivors, p=0.01).

Risk factors associated with increased mortality included pre-existing ESRD requiring hemodialysis (11/13 (86%) p<0.001), vasopressor use (28/48 (58%) p< 0.001), and mechanical ventilation (35/76 (46%) p<0.001). These three variables remained significant when evaluated via multivariate regression analysis (Table 3). Patients with *C. albicans* were also more likely to expire (11 (31%) non-survival vs. 2 (4%) survival, p=0.0003) (Table 5).

**Table 5:** Survival by Candida species ^N(%); p-value<0.05 is considered significant

Candida Species^	Patients Survived N=56	Patients Non-survived N=35	P-value
Candida albicans	2 (4)	11 (31)	0.0003
Candida glabrata	13 (23)	13 (37)	0.15
Candida parapsilosis	13 (23)	12 (34)	0.25
Candida tropicalis	10 (18)	7 (20)	0.79
Candida kefyr	13 (23)	8 (23)	0.99

Comparing our previously reported data from 1997 to 2002, the incidence of *C. glabrata* on culture increased from 13% to 27% during 2006-2012. During this same time period, the frequency of *C. albicans* bloodstream infections decreased from 50% to 40% during 2006-2012 [4].

## Discussion

*Candida* species are the most common cause of fungal infections and rank as the fourth most common cause of bloodstream infections in critically ill patients in the United States, with associated mortality rates as high as 67% [8]. An increasing incidence of *C. glabrata* has been noted, which is generally resistant to fluconazole, the primary drug used to treat *C. albicans*. Accordingly, the Infectious Diseases Society of America (IDSA) guidelines recommend echinocandins as initial therapy for candidemia in non-neutropenic patients [3]. In critically ill patients, the IDSA guidelines advocate for empiric antifungal therapy in patients with risk factors for invasive candidiasis and an unexplained fever [3]. In patients with risk factors [9] and clinical signs of severe sepsis/septic shock, prompt empiric antifungal therapy should be initiated with echinocandins [10]. The literature reports improved outcomes when initiating an echinocandin [11]. This study found that critically ill patients with risk factors for candidemia had increased mortality associated with mechanical ventilation, vasopressor therapy, ESRD, and ≥2 organ failure. Mortality was also more likely in patients who received IAAT compared to AAT.

Reported risk factors linked with invasive candidiasis include central venous catheters, recent antibiotic use, parenteral nutrition administration, renal replacement therapy in critically ill patients, neutropenia, implantable prosthetic devices, and the administration of immunosuppressive agents such as glucocorticoids, anti-rejection agents, or chemotherapy [2,12]. In our patients, candidemia occurred in sicker, critically ill patients, especially when APACHE II > 25 and age > 55.

*C. glabrata* is an increasing problem in critically ill patients. The study site's *C. glabrata* candidemia patients were more commonly older patients, age > 60 years, and had a higher acuity of illness as reflected by the APACHE II > 25. At the time of the study period, *C. glabrata* was not suspected, and empiric therapy was typically initiated with fluconazole. In addition, patients with *C. glabrata *were more likely to have in-hospital mortality when receiving IAAT.

Our overall mortality rate was 38%, which is consistent with the expected mortality for critically ill patients based on our APACHE II score of 25. In non-surgical patients with this APACHE II score, the mortality rate is reported as high as 55%.

Inadequate and delayed antifungal therapy of greater than 48 hours is reported to be associated with prolonged hospitalization, increased healthcare costs, and higher mortality [13]. In 2005, Morrell et al. demonstrated that delaying antifungal therapy beyond 12 hours from the onset of infection increased the risk for mortality by more than twofold [5]. Another study published in 2010 showed a non-significant increase in mortality when antifungal therapy with caspofungin, an echinocandin, was delayed beyond 72 hours [14].

Delays in AAT may occur partly due to a decreased sensitivity (~50%) of blood cultures to detect yeast [15]. In addition, blood cultures in patients with autopsy-proven invasive candidiasis are only positive 38-71% of the time [15]. The time for blood cultures to result positive for *Candida* can be 48-72 hours or longer. Our population of patients demonstrated an increased mortality when AAT was delayed for >72 hours.

This study identified that 37% of patients had concomitant bacteremia, which was not expected. The concomitant candidemia and bacteremia increased the mortality rate to 47% in patients with bacteremia vs. 33% in patients without bacteremia.

Temporal changes in species distributions were previously identified at our hospital. Prior to 2002, the majority of candidemia isolates were *C. albicans* (50%). However, the non-*C. albicans* species have become more prevalent, with *C. glabrata* found in up to 35% of fungal isolates, thus becoming the second leading cause of candidemia since 2000 [4]. Increasingly, azole-resistant *Candida* species, such as *C. glabrata* and *C. krusei*, are becoming prevalent in critically ill patients [16,17]. Hence, if available, the need to empirically cover these pathogens should be determined based on the local prevalence and antifungal susceptibility testing. Comparing our and other's data, it is prudent to initiate an echinocandin empirically when invasive candidiasis is suspected in critically ill patients [3,11].

The limitations of our study include the fact that minimum inhibitor concentration data were not consistently available for *C. glabrata*. In addition, although a peptide nucleic acid fluorescent *in situ* hybridization test became available during the study period to optimize AAT, it was not implemented until 2011. Another limitation of the study is the statistical analysis employed, which does not account for confounding variables that may impact the potential risk factors for candidemia with mortality. Being a retrospective study, the sample size is small and is intended to generate hypotheses.

## Conclusions

Candidemia in the critically ill patient population is a significant problem. It is extremely important to cover for *Candida* species in patients with identified risk factors and an unexplained fever. This study found that *C. glabrata* was more likely in patients requiring hemodialysis for ESRD, requiring vasopressor therapy for hemodynamic support, and having ≥ 2 organ dysfunctions associated with a high APACHE II score. It is imperative to provide an AAT promptly in these patients, especially when candidemia is suspected.
